# Contamination of liposomal bupivacaine during multi-dose usage in a clinical setting evaluated by culture and PCR

**DOI:** 10.3389/fvets.2024.1365679

**Published:** 2024-09-26

**Authors:** Rachel L. Melvin, Jing Wu, Brad S. Bennett, Sara D. Lawhon, Amy Savarino, Artem S. Rogovskyy, Kelley M. Thieman Mankin

**Affiliations:** ^1^Department of Small Animal Clinical Sciences, Texas A&M University College of Veterinary Medicine & Biomedical Sciences, College Station, TX, United States; ^2^Veterinary Medical Teaching Hospital, Texas A&M University College of Veterinary Medicine & Biomedical Sciences, College Station, TX, United States; ^3^Department of Veterinary Pathobiology, Texas A&M University College of Veterinary Medicine & Biomedical Sciences, College Station, TX, United States

**Keywords:** liposomal, bupivacaine, PCR, culture, NOCITA^™^, multi-dose

## Abstract

**Introduction:**

Liposomal bupivacaine, a long-acting local anesthetic, is sold in single-dose vials at a cost of approximately $200/20 mL vial. As many veterinary patients are not dosed an entire vial, the vials have been used for multiple doses at our institution to provide cost savings. Multiple punctures of a vial can lead to increased opportunity for contamination of the contents. This study aims to describe our institutional procedure for multi-dose use of single-dose liposomal bupivacaine vials and to evaluate clinically utilized liposomal bupivacaine for bacterial and fungal contamination using molecular and bacteriological methods.

**Methods:**

The first (Control) and last (Sample B) 0.5 mL from each vial were collected and submitted for bacterial and fungal PCR, anaerobic and aerobic bacterial culture, and opportunistic fungal culture.

**Results:**

All 40 bacterial cultures yielded no growth; Bacterial or fungal DNA was identified in 19 samples (50%). Of the 19 samples in which bacterial or fungal DNA was identified, 10 (52.6%) were from Control, and 9 (47.4%) were from Sample B. PCR does not appear to be useful in detecting bacterial or fungal contamination from liposomal bupivacaine.

**Discussion:**

Results support the aseptic handling protocol described in this article is successful in preventing detectable bacterial and fungal contamination of liposomal bupivacaine vials for up to 7 individual punctures and vials open for up to 5 days.

## 1 Introduction

Liposomal bupivacaine is a local anesthetic designed to gradually release bupivacaine from liposome vesicles to provide analgesia for up to 72 h (1). In dogs, liposomal bupivacaine has been shown to reduce the need for additional postoperative analgesia following Tibial Plateau Leveling Osteotomy (TPLO) when compared to 0.5% bupivacaine hydrochloride (2). The use of liposomal bupivacaine has been associated with a decreased need for postoperative opioids and shorter hospitalization time following exploratory laparotomy (3). An investigation of liposomal bupivacaine following ovariohysterectomy found a decreased need for rescue analgesics and lower pain scores in dogs postoperatively when compared to a bupivacaine splash block (4).

In veterinary medicine, commercially available liposomal bupivacaine (NOCITA^™^, Elanco US Inc.) is sold in 10 mL and 20 mL single-dose vials (13.3 mg/mL) labeled for infiltration of the surgical incision following cranial cruciate ligament surgery in dogs or for nerve block prior to onychectomy in cats (1). NOCITA^™^ is packaged in single-dose vials and dosed at 5.3 mg/kg (0.4 mL/kg) (1). However, the commercially available veterinary suspension is costly, at nearly $10 per mL or nearly $200 per 20 mL vial. Many veterinary patients can only receive a small volume due either to body size and/or the size of the surgical site. Since the medication is sold as a single-dose vial, the owners would be charged for the entire vial. A survey of over 37,036 veterinarians showed that most veterinarians believe that client financial limitations impact the level of care veterinarians are able to provide (5). Anecdotally, the high cost of bupivacaine liposomal suspension has motivated hospitals to either not utilize the medication or engage in off-label multipuncture protocols.

Concerns with multipuncture protocols include bacterial contamination of the drug during the multiple punctures and decreased efficacy of the drug following storage. A study in rats administered bupivacaine liposome injectable suspension showed no significant difference in nociception when the medication was pulled from a single vial and used in a multi-dose fashion over 5 days (6). A different study by Carlson et al. (7) evaluated both bacterial contamination and free bupivacaine when performing multiple punctures of a vial. The group performing that study removed 3 mL aliquots, cultured, and measured the amount of free bupivacaine from 10 different 20 mL liposomal bupivacaine vials, stored either at 24°C or room temperature, daily for 5 days. The investigation found no bacterial growth was detected from any sample over 5 days duration whether stored with refrigeration or at room temperature (7). The free bupivacaine concentration was unchanged for both groups for the first 4 days, however, a mild but significant increase in free bupivacaine was measured on the 5th day (7). This non-clinical study found no bacterial contamination in study vials of liposomal bupivacaine.

Both Callahan et al. (8) and Wallace et al. (9) inoculated bupivacaine liposomal suspensions with bacteria commonly associated with surgical site infections in humans. Callahan et al. (8) showed *Escherichia coli* had significantly greater growth in bupivacaine liposomal suspension when compared to growth in 1% lidocaine or in 0.25% bupivacaine. Wallace et al. (9) showed that when bupivacaine liposomal suspension was inoculated with *Staphylococcus aureus*, *Candida albicans*, *Pseudomonas aeruginosa*, and *E. coli,* only the *P. aeruginosa* and *E. coli* experienced increased growth when compared to 0.5% bupivacaine, suggesting an organism dependent potential for growth.

The goal of this study is threefold: First, to evaluate vials of liposomal bupivacaine that are used in a clinical setting and undergoing multiple punctures for bacterial and fungal contamination using molecular and bacteriological methods. Second, to evaluate the potential utility of PCR testing in the detection of contamination of liposomal bupivacaine vials during multi-puncture use. Third, to describe our institutional procedure of using single-dose liposomal bupivacaine vials as multi-dose vials.

## 2 Materials and methods

Samples of liposomal bupivacaine were collected from 20 mL vials of NOCITA^™^ (Elanco USA Inc., Greenfield, IN, United States). The liposomal bupivacaine was used on clinical patients (cats and dogs) at our institution and the vials were handled as per our hospital’s standard protocol with the exceptions of the first and last 0.5 mL of liposomal bupivacaine which were collected for the study.

The handler first washed their hands and put on exam gloves that were then drenched with 70% isopropyl alcohol. The liposomal bupivacaine vial, needle, and syringe were then cleaned with 70% isopropyl alcohol and placed in the laminar flow hood in our institution’s pharmacy (NuAire Lab Equipment, Plymouth, MN, United States). Working inside the flow hood, the metal cap of the vial was removed, and the first needle puncture of each vial was used to withdraw 0.5 mL of liposomal bupivacaine (Control), individual volumes for in-hospital use were then withdrawn using a sterile syringe and needle. Control sample and the liposomal bupivacaine vial were then immediately placed in a refrigerator at 4°C. The Control sample was picked up and transported to the laboratory. The liposomal bupivacaine vial was then only removed from the refrigerator for patient medication acquisition. All aliquots for patient use were withdrawn using the same technique under the laminar flow hood. A record was maintained of how much volume was removed from each vial and how much should remain. When approximately 0.5 mL of liposomal bupivacaine remained in the vial, no more was withdrawn; the last approximately 0.5 mL was left in the vial until submission, this was Sample B. Sample B was refrigerated at 4°C until it was picked up and transported to the laboratory. This protocol is the institutional procedure for using single dose liposomal bupivacaine vials as multi-dose vials, with the exception of the withdrawal of Control and Sample B.

For each vial, the following data was recorded on a data collection sheet: the date and time of puncture for Control collection, the date and time, the volume withdrawn, the remaining volume (calculated) in the vial, and the intended patient for each subsequent withdrawal, and the date and time of Sample B collection. Control and Sample B underwent bacterial and fungal testing including bacterial and fungal PCR, anaerobic and aerobic bacterial culture, and opportunistic fungal culture. The standard techniques for samples submitted to our institution’s clinical microbiology lab were utilized.

All cultures were performed according to standard laboratory protocol. A small amount of each sample was plated onto Brucella Blood agar with Hemin, and Vitamin K (BRU; Hardy Diagnostics, Santa Maria, CA, United States), Columbia CNA with 5% Sheep Blood agar (CNA; Becton Dickinson and Company (BD&C), Franklin Lakes, NJ, United States), and Brain Heart Infusion broth (BD&C) with Oxyrase (Oxyrase, Mansfield, OH, United States) for anaerobic culture and onto Trypticase Soy Agar with 5% Sheep Blood (TSA II; BD&C), Chocolate Agar plate (BD&C), MacConkey plate (BD&C), CNA and Tryptose broth (BD&C) for aerobic culture. The plated samples were incubated in an anaerobe jar with an anaerobic gas generating sachet (AnaeroPack; Mitsubishi Gas Chemical America, Inc., New York, NY, United States) at 37°C for anaerobic conditions and in 6.1% CO_2_ at 37°C for aerobic cultures, respectively, and observed daily for 5–6 days for evidence of bacterial growth. Another small amount of each sample was plated onto Potato Dextrose agar (Hardy Diagnostics) and Sabouraud Dextrose agar (Hardy Diagnostics) then incubated at room temperature and observed daily for 21 days for evidence of fungal growth.

PCR followed by Sanger sequencing of the resultant PCR products was performed for identification of bacterial and fungal DNA. DNA from each sample was extracted using a QIAamp^®^ PowerFecal^®^ Pro DNA Kit (Qiagen, Germantown, MD, United States). PCR was performed using AmpliTaq^™^ DNA Polymerase (Applied Biosystems^™^; ThermoFisher Scientific, Waltham, MA, United States) was used at a final concentration of 1 U in a total reaction volume of 50 μL consisting of a final concentration of the supplied PCR Buffer at 1X concentration with 2.5 mM MgCl_2_, 0.2 mM of each deoxynucleoside tripohsphate, and 0.3 μM each of the forward and reverse primers were used for amplification. PCR was performed using a dual-block DNA Engine thermal cycler (BioRad, Hercules, CA, United States) with an initial 3-min denaturation step at 94°C, followed by 40 cycles consisting of a 30-s denaturation step at 94°C, a 30-s annealing step at 56°C, and a 30-s extension at 72°C, finishing with a final extension step for 7 min at 72°C. For detection of bacterial DNA, the sequence of the forward primer, 515F, was 5′-GTGCCAGCAGCCGCGGTAA-3′, and the sequence of the reverse primer, 13R, sequence was AGGCCCGGGAACGTATTCAC-3′ (10). For detection of the fungal DNA, the primers ITS1 5′-TCCGTAGGTGAACCTGCGG-3′ and ITS4 5′-TCCTCCGCTTATTGATATGC-3′ were used (11). The resultant PCR products were submitted to a commercial laboratory for Sanger sequencing (Eton Bioscience, Inc., San Diego, CA, United States). Sequence results were then compared to GenBank and top matches were reported. Results were categorized as bacterial or fungal DNA identified (DNA with greater than 95% identity), no similarity (DNA with <95% identity), or no bacterial or fungal DNA detected.

Descriptive statistics were performed for the number of punctures, the “duration open” (time between first puncture and last puncture), bacterial and fungal culture results, and bacterial and fungal PCR results.

## 3 Results

Data was collected for 22 vials; 2 vials were excluded from analysis as one of the samples (Control or Sample B) was not submitted for testing. Statistical analysis was performed on data collected for the remaining 20 vials (Table 1). All 20 vials were punctured between 3 and 7 times for withdrawal of liposomal bupivacaine with a mean of 5.00 +/− 1.26 individual punctures. The “duration open” ranged from 0.02 h to 120.87 h, the mean time a vial was opened was 33.43 +/− 33.51 h.

**Table 1:** Data for all 20 vials.

Vial ID	Number of punctures per Vial	Time (hours) between the 1st and last withdrawal	Sample ID	Culture results			PCR results		
				Aerobic	Anaerobic	Fungal	Identity (%)	Categorization	Organism order or genus/species
1	7	25.50	Control	NG	NG	NG	98.4	Bacterial DNA Identified	Enterobacterales
			B	NG	NG	NG	100	Bacterial DNA Identified	Enterobacterales
2	5	22.67	Control	NG	NG	NG	100	Bacterial DNA Identified	Enterobacterales
			B	NG	NG	NG	98.51	Bacterial DNA Identified	Enterobacterales
3	7	26.90	Control	NG	NG	NG	91.49	No Similarity	n/a*
			B	NG	NG	NG	95.32	Bacterial DNA Identified	*Rhizobium*
4	5	73.43	Control	NG	NG	NG	100 100	Bacterial DNA Identified	Enterobacterales *Budvicia*
			B	NG	NG	NG	98.98	Bacterial DNA Identified	Enterobacterales
6	4	24.42	Control	NG	NG	NG	99.59	Bacterial DNA Identified	Enterobacterales
			B	NG	NG	NG	92.12	No Similarity	n/a*
7	4	23.50	Control	NG	NG	NG	99.6	Bacterial DNA Identified	Enterobacterales
			B	NG	NG	NG	91.67	No Similarity	n/a*
8	4	0.75	Control	NG	NG	NG	98.26	Bacterial DNA Identified	Enterobacterales
			B	NG	NG	NG	98.66	Bacterial DNA Identified	*Ralstonia*
9	5	24.25	Control	NG	NG	*Sarocladium strictum*	91.76	No Similarity	n/a*
			B	NG	NG	NG	84.26	No Similarity	n/a*
10	4	94.65	Control	NG	NG	*Epicoccum* spp.	90.39	No Similarity	n/a*
			B	NG	NG	NG	100	Bacterial DNA Identified	Enterobacterales
11	3	0.85	Control	NG	NG	NG	Poor Band	No Similarity	n/a*
			B	NG	NG	NG	96.55	Bacterial DNA Identified	Enterobacterales
12	5	23.15	Control	NG	NG	NG	99.57	Bacterial DNA Identified	Enterobacterales
			B	NG	NG	NG	Poor Band	No Similarity	n/a*
13	6	24.20	Control	NG	NG	NG	Poor Band	No Similarity	n/a*
			B	NG	NG	NG	99.72	Bacterial DNA Identified	*Staphylococcus auricularis*
14	6	120.87	Control	NG	NG	NG	99.55	Bacterial DNA Identified	Enterobacterales
			B	NG	NG	NG	Poor Band	No Similarity	n/a*
15	6	1.17	Control	NG	NG	NG	n/a*	n/a*	n/a*
			B	NG	NG	NG	n/a*	n/a*	n/a*
16	4	26.20	Control	NG	NG	NG	85	No Similarity	n/a*
			B	NG	NG	NG	98.77	Bacterial DNA Identified	*Methyloversatilis*
17	7	25.40	Control	NG	NG	NG	99.82	Fungal DNA Identified	*Saccharomyces cerevisiae*
			B	NG	NG	NG	90.35 89.91	No Similarity	n/a*
18	4	87.83	Control	NG	NG	NG	88.83	No Similarity	n/a*
			B	NG	NG	NG	91.61 91.61	No Similarity	n/a*
19	3	0.02	Control	NG	NG	NG	98.22	Bacterial DNA Identified	*Anaerococcus*
			B	NG	NG	NG	94.02 94.02	No Similarity	n/a*
21	5	24.33	Control	NG	NG	NG	85.54	No Similarity	n/a*
			B	NG	NG	NG	Poor Band	No Similarity	n/a*
22	6	18.53	Control	NG	NG	NG	No Band	No Bacterial DNA Detected	n/a*
			B	NG	NG	NG	Poor Band	No Similarity	n/a*

*n/a denotes non-applicable; NG denotes no bacterial or fungal growth.

Forty samples from 20 vials were submitted for anaerobic and aerobic culture, no colonies grew for any of the samples (Table 1). Two samples (5%) grew fungal organisms on culture - *Sarocladium strictum* and *Epicoccum* spp. Both fungal organisms were grown from Control and the organism did not grow on Sample B from either vial.

PCR was performed on 38 samples from 19 vials. One vial was excluded from PCR analysis as the sample did not have sufficient volume after culture setup - leaving 19 vials (38 samples) available for analysis of PCR results (Table 1). Results were categorized as bacterial or fungal DNA identified (DNA with greater than 95% identity), no similarity (DNA with <95% identity), or no bacterial or fungal DNA identified (Table 2). Samples categorized as no similarity were samples in which DNA material was detected but could not be identified, i.e., non-identifiable DNA. Bacterial or fungal DNA was identified in 19 samples (50%) from 15 different vials. Non-identifiable DNA in 18 samples (47.4%) from 15 different vials. In only one sample (2.6%) from 1 vial was no evidence of bacterial or fungal DNA found on PCR. Of the 19 samples in which bacterial or fungal DNA was identified, 10 (52.6%) were from Control, and 9 (47.4%) were from Sample B.

**Table 2:** PCR results were categorized based on the percent identity with bacterial or fungal DNA sequences in the GenBank database.

	*n*	DNA identified		No similarity		No DNA detected
		100%	95–99%	<95%	Poor	
Control	19	2	8	6	2	1
		10.53%	42.11%	31.58%	10.53%	5.26%
Sample B	19	2	7	6	4	0
		10.53%	36.84%	31.58%	21.05%	0.00%
Total	38	4	15	12	6	1
		10.53%	39.47%	31.58%	15.79%	2.63%
		19		18		1
		50.00%		47.37%		2.63%

Bacterial or fungal DNA was considered identified if identity with a bacterial or fungal sequence in the GenBank database was above 95%. Those below 95% identity were not considered to have no similarity with any bacterial or fungal DNA in the database—i.e. non-identified DNA. PCR was unable to detect any DNA sequence in a single sample.

PCR of 38 samples identified DNA from 6 different bacteria and 1 yeast (Table 3). A member of Enterobacterales was identified in 13 samples (34.2%); 8 times from Control (42.1%) and 5 times from Sample B (13.2%). *Saccharomyces cerevisiae*, *Anaerococcus* spp., *Rhizobium* spp., *Ralstonia* spp., *Methyloversatilis* spp., and *Budvicia* spp. were each identified in 1 sample (2.6%) each (Table 3). Four times, the same organism, a member of Enterobacterales, was identified by PCR in both Control and Sample B from the same vial. Once, PCR identified the DNA from 2 separate organisms, a member of Enterobacterales and a *Budivicia* spp. in the same sample.

**Table 3:** PCR identified DNA from 6 different bacteria and 1 yeast species in 19 samples.

	Bacterial DNA identified			
	Control		Sample B	
	95–99.99%	100%	95–99.99%	100%
*Enterobacterales*	6	2	3	2
*Anaerococcus*	1	0	0	0
*Rhizobium*	0	0	1	0
*Ralstonia species*	0	0	1	0
*Methyloversatilis*	0	0	1	0
*Budvicia*	0	1	0	0
*Saccharomyces cerevisiae*	1	0	0	0

DNA from a single bacterial or fungal organism was identified in 18 samples and DNA from 2 bacterial organisms was identified in a single sample. Members of Enterobacterales were the most commonly identified.

## 4 Discussion

This study investigated the contamination risk when using liposomal bupivacaine off-label in a multi-dose fashion in a clinical setting and the utility of PCR in assessing bacterial and fungal contamination of liposomal bupivacaine. We found no evidence of bacterial or fungal contamination of the suspension through multi-dose use; however, PCR was not found to be a useful diagnostic in detecting bacterial or fungal contamination.

All vials were punctured multiple times for patient dosing, ranging from 3 to 7 punctures per vial. Vials were opened for variable durations ranging from 1.2 min (19 mL was withdrawn for patient use at the same time as the Control collection) to approximately 5 days. The number of punctures and duration open were not controlled as the experiment was performed in a clinical setting to allow for analysis of associated risk with each variable. This contrasts previous studies of multi-dose use of liposomal bupivacaine in which aliquots were only withdrawn for laboratory tests (7) or vials were experimentally contaminated with various organisms (8, 9). The only other study that evaluated multi-dose use of liposomal bupivacaine solely analyzed the analgesic efficacy of the medication (6), no microbiology testing was performed.

Aerobic and anaerobic cultures have been shown to be effective in studies of purposeful contamination of liposomal bupivacaine (8, 9). In the present study, aerobic and anaerobic cultures resulted in no growth over 5–6 days for any of the 40 samples submitted for testing. The finding of negative cultures in the present study is consistent with a non-clinical study by Carlson *et al* who demonstrated no bacterial growth after repeated withdrawals from liposomal bupivacaine over 5 days (7). In the present study, fungal culture did result in growth from 2 samples. Both positive fungal cultures were from Control aliquots and no fungal growth was observed from either corresponding Sample B, nor was fungal DNA detected on PCR testing of either sample. Therefore, contamination of the samples post-collection or culture media during plating is suspected. Carlson et al. (7) similarly cultured an *Aspergillus* spp. that grew from a control sample and was suspected to represent environmental contamination. These fungal and bacterial culture results suggest the currently used liposomal bupivacaine aseptic handling protocol is successful in preventing contamination of the vial for up to 7 individual vial punctures for medication withdrawal in vials open for up to 5 days.

Although bacterial or fungal DNA was identified in 50% of the samples, no growth was seen on anaerobic or aerobic culture, suggesting the DNA detected on PCR represents fragmented or non-viable bacterial or fungal organisms. Two samples had growth on fungal culture, these were suspected to be due to contamination, as discussed above. No fungal DNA was detected on the fungal PCRs performed on the same samples as the positive fungal cultures. The bacterial and fungal DNA identified by PCR were presumed to be nonviable as there was no growth on either aerobic bacterial, anaerobic bacterial, or fungal culture, however, the greatest disadvantage of PCR is its inability to distinguish between live and dead cells/viable and non-viable DNA (12). Future studies could utilize viability or rapid viability PCR (vPCR or rvPCR), a PCR technique that involves the addition of a chemical to a sample prior to PCR processing that selectively enters damaged cells to bind the DNA and prevent amplification (13, 14), to confirm that identified DNA represent nonviable organisms.

Callahan et al. (8) and Wallace et al. (9) both showed that *E. coli*, a bacteria within the Enterobacterales order, had greater growth in liposomal bupivacaine suspensions than in saline, 1% lidocaine, 0.25% bupivacaine, 0.5% bupivacaine, or propofol. The majority of DNA identified in our samples was from members of Enterobacterales. If the DNA source was contamination of the vial during the withdrawal process, one would expect DNA to be identified more frequently in Sample B aliquots than in Control aliquots. Further, we would expect contamination of the vial to result in positive bacterial culture. Greater than 50% of the samples in which bacteria was identified were from the Control sample and no samples had growth on aerobic or anaerobic culture, suggesting that the identified DNA was present prior to the first puncture of the vial. Based on the fact that Control and Sample B had bacteria detected on PCR at almost equal rates (52.6% vs. 47.4%, respectively) and the fact that no bacteria were grown on bacterial culture from any samples, we expect that the DNA detected on PCR originates from equipment or material during the manufacturing process of the liposomal bupivacaine.

The main limitation of this study is patients in which the liposomal bupivacaine was used were not evaluated for surgical site infection and/or complication as it was beyond the scope of this study. It is also important to note that each withdrawal/puncture of the liposomal bupivacaine vial was performed with aseptic technique utilizing a laminar flow hood. Utilization of a laminar flow hood would not be expected to be easily achievable in general practice. Future research should investigate the efficacy of an aseptic handling protocol in a clean area (not a laminar flow hood) when multi-dosing liposomal bupivacaine in hospitals without a laminar flow hood. Follow-up studies could also investigate the incidence of surgical site infections and other complications between incisions instilled with liposomal bupivacaine used in a single dose versus multidose fashion.

The present study does not support the usefulness of PCR in detecting small amounts of bacterial or fungal DNA indicating bacterial or fungal contamination of liposomal bupivacaine vials as only 1 sample (Vial 22, Control) was free of bacterial or fungal DNA on PCR testing (truly negative for DNA material), no bacteria were cultured from any sample, and the PCR results for Control and Sample B were not notably different. We suspect that during the creation and processing of liposomal bupivacaine, DNA or DNA fragments are incorporated into the sterile medication, producing positive PCR results. In this study, PCR was not helpful in the identification of possible pathogenic bacterial or fungal contamination as suspected non-viable bacterial and fungal DNA were amplified and identified. This study also supports multiple withdrawals from a single-use liposomal bupivacaine vial over 0–5 days using the aseptic technique described does not result in detectable bacterial contamination.

## Data Availability

The original contributions presented in the study are included in the article/supplementary material, further inquiries can be directed to the corresponding author.
